# Substitution of histidine 30 by asparagine in manganese superoxide dismutase alters biophysical properties and supports proliferation in a K562 leukemia cell line

**DOI:** 10.1007/s00249-021-01544-2

**Published:** 2021-05-21

**Authors:** Rosalin Bonetta, Gary J. Hunter, Chi H. Trinh, Tomasz Borowski, Anthony G. Fenech, Maria Kulp, Leandro C. Tabares, Sun Un, Thérèse Hunter

**Affiliations:** 1 Centre of Molecular Medicine & Biobanking, University of Malta, Msida, Malta; 2 Department of Physiology and Biochemistry, Faculty of Medicine and Surgery, University of Malta, Msida, Malta; 3 Astbury Centre for Structural Molecular Biology, Institute of Molecular and Cellular Biology, University of Leeds, Leeds, UK; 4 Jerzy Haber Institute of Catalysis and Surface Chemistry, Polish Academy of Sciences, Krakow, Poland; 5 Department of Clinical Pharmacology and Therapeutics, Faculty of Medicine and Surgery, University of Malta, Msida, Malta; 6 Department of Chemistry, Tallinn University of Technology, Tallinn, Estonia; 7 Institute for Integrative Biology of the Cell (I2BC), Université Paris-Saclay, CEA, CNRS, 91198 Gif-sur-Yvette, France; 8 Present Address: Barts and the London, School of Medicine and Dentistry, QMUL, Victoria, Malta

**Keywords:** Proteins, Superoxide dismutase, Structural modelling, Spectroscopy, Biophysics, X-ray crystallography

## Abstract

We have generated a mutant of *C. elegans* manganese superoxide dismutase at histidine 30 by site-directed mutagenesis. The structure was solved at a resolution of 1.52 Å by X-ray crystallography (pdb: 6S0D). His30 was targeted, as it forms as a gateway residue at the top of the solvent access funnel to the active site, together with Tyr34. In the wild-type protein, these gateway residues are involved in the hydrogen-bonding network providing the protons necessary for the catalytic reaction at the metal center. However, biophysical characterization and cell viability experiments reveal that a mutation from histidine to asparagine in the H30N mutant modifies metal selectivity in the protein, favoring the uptake of iron over manganese in minimal media conditions, alters active-site coordination from the characteristic trigonal bipyramidal to octahedral geometry, and encourages cellular proliferation in K562 cells, when added exogenously to the cells.

**Supplementary Information:**

The online version contains supplementary material available at 10.1007/s00249-021-01544-2.

## Introduction

**Scheme 1:**
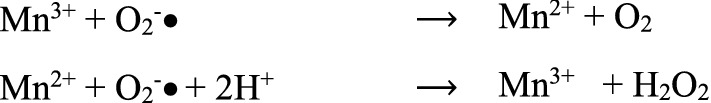
Dismutation by SOD

Eukaryotic manganese superoxide dismutase (SOD, EC 1.15.1.1) is a nuclear-encoded protein that is synthesized with an N-terminal leader sequence that targets it to the mitochondrion. The mature homotetrameric MnSOD resides in the mitochondrial matrix where it provides protection against the superoxide radicals generated by the electron transport chain (Sheng et al. 2014). The nature of the MnSOD catalytic reaction illustrates the dual role this enzyme plays as both an antioxidant and a modulator of the redox status. During catalysis, the cyclic reduction and oxidation of the active site manganese cofactor is accompanied by the dismutation of two superoxide anions to molecular oxygen and hydrogen peroxide (Scheme 1) (Bull et al. 1991; McAdam et al. 1977).

Consequently, the rate at which the superoxide is removed and the subsequent release of hydrogen peroxide into the immediate environment contribute to the transient redox status of the cell. Since cellular membranes are highly permeable to hydrogen peroxide and superoxide can move from the inter-membrane space to the cytoplasm via voltage dependent anion channels (VDAC) (Han et al. 2003), changes in the ratio of superoxide to hydrogen peroxide have far-reaching consequences for the cell.

Furthermore, the degree of metalation with the manganese cofactor during the folding of nascent protein influences the activity of MnSOD and appears to be optimized by HSP60 (Bie et al. 2016; Hunter et al. 2015; Hunter and Hunter 2013; Magnoni et al. 2014).

**Scheme 2:**
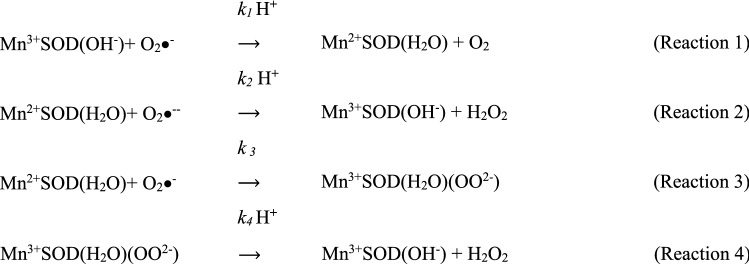
Mechanism of catalysis of MnSOD

The *C. elegans* nematode produces two distinct mitochondrial MnSODs; MnSOD-2 and MnSOD-3. *Mnsod-2* is expressed constitutively throughout all the life stages of nematode while the *Mnsod-3* gene is a target of DAF-16/FOXO and is induced during the dauer stage in response to stressful environmental conditions (Honda and Honda 1999). Interestingly, this protein is also produced by the longevity mutants DAF-2 and AGE-1, which have altered insulin/IGF-1 signaling. Despite the high structural homology amongst the active sites of studied eukaryotic MnSODs, there are differences in their kinetic profiles that may influence the cellular response to the redox status. The MnSOD catalytic mechanism has been described by the McAdam scheme as four reactions that occur via two simultaneous pathways, the outer and the inner-sphere pathways (McAdam et al. 1977) (Scheme 2).

The outer-sphere pathway, represented in Scheme 2 by reaction 1 and 2, reduces superoxide to hydrogen peroxide instantaneously under normal conditions of superoxide levels. When the superoxide levels are elevated, reaction 3 of the inner-sphere pathway results in the formation of the Mn-peroxy complex that inhibits the enzyme. The rate of dissociation of this complex and the release of the hydrogen peroxide product is described by *k*_*4*_. The gating ratio *k*_*2*_*/k*_3_ is used to determine the favored pathway and whether the production of hydrogen peroxide is very fast (*k*_*2*_*/k*_*3*_ > 1) or whether the enzyme is product-inhibited (*k*_*2*_*/k*_*3*_ < 1) (Hearn et al. 1999; Hsu et al. 1996). Known prokaryotic MnSODs tend to function via the instantaneous outer-sphere pathway (Sheng et al. 2012).

While human and *C. elegans* MnSOD are structurally very similar, the product dissociation constant (k_4_ 120 s^−1^) of the human enzyme is lower than that of the *C. elegans* MnSOD-3 (*k*_*4*_ 300 s^−1^) (Hunter et al. 2015). This may be a mechanism adopted by human cells to prevent the production of high levels of hydrogen peroxide when superoxide levels are elevated, reducing any signaling response to the hydrogen peroxide (Abreu and Cabelli 2010). His30 and Tyr34 are gateway residues, positioned at the top of the solvent access funnel to the active site. They participate in the hydrogen-bonding network that provides the protons necessary for the catalytic reaction at the metal center. By studying the effect of MnSOD-3 harboring His30 mutations on a K562 leukemia cell line, we tested whether the activity of MnSOD is a factor that controls the molecular switch between cellular proliferation and apoptosis.

The involvement of MnSOD in cancer development, progression, and prevention has been a contentious one as varying levels of MnSOD expression and activity have been associated with different cancer types during different stages of progression. Low levels of the enzyme appear to support transformation of normal cells, most likely as a consequence of ineffective antioxidant protection during early stage carcinogenesis (Dhar et al. 2011). However, the expression of *Mnsod* has been reported to increase during the establishment of an aggressive, invasive cancer phenotype (Connor et al. 2007). The activity of MnSOD also appears to determine the tumor-suppressor or tumor-promoter character of the enzyme (Dhar and St Clair 2012).

## Materials and methods

### Reagents and cells

All general-purpose chemicals and buffers were obtained from Sigma–Aldrich (Germany) and VWR International (Radnor, USA) and bacteriological media was from Oxoid (Basingstoke UK). The Quikchange II XL site-directed mutagenesis kit was supplied by Agilent Technologies (Santa Clara, Ca). The PureLink HQ plasmid mini prep kit and the Alexa Fluor^®^ 488 Annexin V/Dead cell Apoptosis Kit were purchased from Invitrogen (Waltham, MA). The Caspase-GIo 3/7, 8 and 9 Assay kits and the CellTiter-Glo^®^ assay kit were from Promega (Madison, WI). All the oligonucleotides were synthesized by Bioneer (South Korea).

### Site-directed mutagenesis

The cDNA of the *C. elegans* MnSOD-3 (protein is designated as MnSOD-3WT throughout), previously cloned into a pTrc99A expression system (Hunter et al. 1997), served as the template for site-directed mutagenesis utilizing the QuikChange II XL system. The following oligonucleotide pairs were used to replace histidine 30 with asparagine or phenylalanine, respectively (mutagenic bases are underlined).

H30N Forward: 5′- CTTCATCATCAAAAGAATCATGCCACCTACGTG-3′, H30N Reverse: 5′-CACGTAGGTGGCATGATTCTTTTGATGATGAAG-3′, H30F Forward: 5′-GCTTCATCATCAAAAGTTTCATGCCACCTACGTGAAC-3, H30F Reverse: 5′-GTTCACGTAGGTGGCATGAAACTTTTGATGATGAAGC-3’.

Positive mutants were confirmed by sequencing using PKPro-5 (CTCGTATAATGTGTGGAATTGTGAGCGG) and PKTerm-5 (CCTGACCCCATGCCGAACTCAGAAG) sequencing primers. The mutants of MnSOD-3WT were designated H30N and H30F.

### Protein purification and characterization

The synthesis of recombinant MnSOD was carried out in *E. coli* strain, OX326A (Δ*sodA*, Δ*sodB*), kindly provided by Prof. Steinman, Albert Einstein College of Medicine, New York (Steinman 1992). Both the production and purification of the all the proteins used in this study were performed as previously described (Trinh et al. 2008). Concentrations of pure protein were measured by absorbance at 280 nm using the extinction coefficient of 43,340 M^−1^ cm^−1^. The manganese and iron content of the purified protein was determined by GF-AAS or ICP-MS. Superoxide dismutase activity was measured spectrophotometrically as described by McCord and Fridovich and Ysebaert-Vanneste and Vanneste, whereby cytochrome *c* serves as the detector and xanthine–xanthine oxidase as the superoxide generator (McCord and Fridovich 1969; Ysebaert-Vanneste and Vanneste 1980). The inhibition of MnSOD activity by the substrate analog, sodium azide, was measured by the cytochrome *c* assay following 10 min incubation at room temperature with 4 mM or 10 mM sodium azide. Optical absorption spectra of MnSOD and H30N (3 mg mL^−1^ in 10 mM Tris.Cl pH 8.0) in the absence and presence of 100 mM sodium azide were collected using a Beckman DU7500 diode array spectrophotometer.

### Crystallization, data collection, structure determination, and refinement

The hanging-drop vapor diffusion method was used to grow crystals from drops composed of 2 µL protein (8 mg/mL) and 2 µL reservoir solution equilibrated at 18˚C. The optimal reservoir solution (500 µL) for H30F and H30N consisted of 0.1 M bicine pH 9.0 and 2.8 M ammonium sulfate. Crystals grew to 350 × 300 × 300 µm and were flash-cooled in liquid nitrogen liquor after soaking for 30 s in solution mother liquor containing 25% glycerol as cryoprotectant solution before mounting on loops for data collection. X-ray diffraction data were recorded at 100 K on stations I02, and I04, respectively, at the Diamond Light Source Ltd using a Dextris Pilatus 6 M-F detector. Data were integrated at 1.6 and 1.52 Å resolution, respectively, using the program XIA2. Data reduction and any following calculations were carried out using the CCP4 program suite (Collaborative Computational Project, Number 4, 1994). Both crystals had a space group of P4_1_2_1_2, with unit-cell parameters *a* = *b* = 81.5 Å, *c* = 138.7 Å for H30F and *a* = *b* = 82.5 Å, *c* = 135.2 Å for H30N. Two subunits per asymmetric unit are present in each protein structure, comprising all amino acids in the sequence.

The H30F and H30N protein structures were determined using the 293 K native MnSOD-3WT structure as the search model, by difference Fourier. Model building and refinement were performed using Coot (Emsley et al. 2010) and REFMAC5 (Vagin et al. 2004), respectively. Non-crystallographic symmetry averaging was not employed during refinement. TLS parameters (Winn et al. 2001) based on a single-group TLS model for each monomer were calculated using the TLS Motion Determination server (http://skuld.bmsc.washington.edu/~tlsmd/) and refined in REFMAC5 during the final stages of refinement. The final structures of H30F and H30N were refined to *R* = 18.8% and R free = 20.6%, and *R* = 17.0% and R free = 21.9%, respectively, at 100 K.

### Circular dichroism spectroscopy

Protein stability was assessed by CD measurements using a Chirascan™ CD spectrometer (Applied Photophysics, Leatherhead, UK) equipped with a Peltier temperature 4-cell auto-changer cell holder. Far-UV (180–260 nm) CD spectra of the proteins (0.3 mg mL^−1^ in 10 mM potassium phosphate buffer pH 7.8) were recorded in a cuvette of 0.1 cm path length with a 4.3 nm bandwidth. To calculate the unfolding transition temperature (T_m_), the proteins were heated from 5 to 90 °C with a 2 °C step size and ± 0.2 °C tolerance for the thermal scans. The collected data were corrected for solvent contributions due to the phosphate buffer. The signal at 222 nm at different temperatures was used to determine the melting temperature after normalization of the curves. The CD curves were normalized using the equation ${\mathrm{CD}}_{\mathrm{norm}}= \frac{CD\left(T\right)-FU(T)}{FF\left(T\right)-FU(T)}$, whereby the CD_(norm)_ is the normalized CD curve and FF(T) and FU(T) are the asymptotes of the original CD curves at high and low asymptotes, respectively. Data were fitted to an asymmetric (5^th^ parameter) least squares fit.

### HFEPR spectroscopy

High-field, continuous-wave electron paramagnetic resonance (HFEPR) spectroscopy was applied to determine the coordination center of MnSOD-3WT and the H30N mutant protein. The HFEPR spectrometer has been described in detail elsewhere (Un et al. 2001). Field calibration was based on a Mn(II)-doped MgO standard sample (*g* = 2.000101) (Burghaus et al. 1993). The absolute error in field measurements was 1 G (0.1 mT) or 0.0001 in g. All spectra were obtained using 10 G modulation under non-saturating conditions at 285 GHz with 5 G resolution at 23 K. The temperature was chosen to maximize the signal arising from the *m*_*s*_ =  − 1/2 1/2 transition at 285 GHz (Un et al. 2004). The quality of spectra could be reproduced reliably twelve times and double integrated for three separate experiments. Samples (500 µL at 200 µM) were mixed with 10 mM sodium dithionite (30 µL) to reduce the metal center to the Mn^2+^ state. The samples were subsequently washed twice with 20 mM Tris.Cl pH 7.8 and 5 mM EDTA followed by a final wash with 20 mM Tris.Cl pH 7.8. 500 µl of sample were loaded in the EPR tubes (2 ml Nalgene plastic cryogenic tubes, immediately frozen in liquid nitrogen and sored at −80 ºC to prevent reoxidation.

### Molecular dynamics simulations

MD simulations were performed by AMBER 16 (Case et al. 2005). The force field parameters for amino acid residues were ff14SB. Our parameters represented Mn and Fe in the 2^+^ state using the 12–6-4 LJ-type non-bonded model AMBER force field (frcmod.ions234lm_1264_tip3p). The TIP3P water solvent model with a thickness of at least 10 Å was used, and chlorine ions were added as counter-ions. The system, including an explicit solvent model, was simulated with a periodic boundary condition, and the particle mesh Ewald method was used to process long-range electrostatic interactions. A cut-off of 10 Å was used for nonbonding interactions. The complements of the hydrogen atoms, not be observed by X-ray crystallographic analysis, were performed by the tleap module of AmberTools. First, the structural minimizations were conducted for the initial structures. The minimization was done in three steps. This involved the first two steps consisting of 5000 minimization steps with harmonic constraint on protein atoms coordinates (force constant of 500 and 10 kcal/mol*A, respectively) and a third step consisting of 10 000 minimization steps with no constraints. After the minimizations, MD simulations of the protein were performed for 80 ns at a constant temperature (300 K) and constant pressure (1 bar), using Langevin dynamics algorithm, a time-step of 2 fs and non-bonded cut-off of 8 Å. Analysis was carried out using VMD (Humphrey et al. 1996).

### Cell culture

The human K562 chronic myeloid leukemia cell line, received through collaboration with the Erasmus Medical Center, Rotterdam, was originally acquired from ATCC (cell line no. ATCC^®^ CCL-243™). The cells were stored in liquid nitrogen, with a low passage number (< 10) and tested negative for *Mycoplasma* infection. K562 cells were cultured under normal conditions (5% CO_2_) at 37 °C in RPMI 1640 medium supplemented with 10% fetal bovine serum (FBS) and 1% Penicillin–Streptomycin solution (5000 units/mL of penicillin and 5000 µg/mL of streptomycin).

### Cell viability

K562 cells in serum-free RPMI media were plated at 5 × 10^3^ or 3 × 10^3^ cells/well in white opaque 96-well plates and incubated for 24 h at 37 °C and 5% CO_2_ in a humidified atmosphere before the addition of MnSOD protein (MnSOD-3WT, H30N or H30F). A control supplemented with only Tris.Cl (pH 8.0) was prepared in parallel. The cells were incubated further for 24-, 48-, and 72-h intervals in the presence of increasing amounts of protein. Cell viability was monitored at the end of each interval by the CellTiter-Glo reagent that generates a luminescent signal that is proportional to the amount of ATP present. Luminescence was recorded using a Mithras LB 940 Multimode Microplate Reader (Berthold Technologies) at 0.5 s per well integration time. All measurements were done in triplicate for a minimum of three independent experiments. In a similar manner, cells were also treated with catalase to confirm that the observed effect was in fact due to the hydrogen peroxide product of the SOD reaction. The 96-well plates were seeded with 5,000 K562 cells per well in serum-starved RPMI medium and incubated for 24 h. Cells were then treated with 10 or 20 μg MnSOD-3WT, 10 or 20 μg MnSOD-3WT together with 1 μg catalase (3 Units) and 1 μg catalase alone. The plates were incubated for 24, 48, and 72 h. Cytotoxicity was evaluated using the CellTiter-Glo^®^ luminescent cell viability assay. Measurements were performed in triplicate.

### Determination of cell death by flow cytometry and fluorescence microscopy

Apoptosis and necrosis were analyzed by fluorescence double staining with Annexin V/Propidium iodide (PI) (Vermes et al. 1995) using the Alexa Fluor^®^ 488 Annexin V/Dead cell Apoptosis Kit (Invitrogen). Approximately 1 × 10^6^ cells under serum-starved conditions and supplemented with MnSOD-3WT for 48 h, were collected, washed with PBS and resuspended in 100 µL 1X Annexin-binding buffer. Untreated K562 cells served as the control. A positive control for apoptosis was prepared by treating cells with benzo(*a*)pyrene (20 μM) for 72 h. The cells were stained with Alexa Fluor^®^ 488 Annexin V (5 µL) and PI (1 µL of 100 μg mL^−1^) and incubated in the dark for 15 min at room temperature. Data were collected using a BD FACSCalibur™ flow cytometer (BD Biosciences, USA) and analyzed using the BD CellQuest™ Pro software. Gating of the cells was based on a cell-associated fluorescence of 10,000 events per sample. Excitation was carried out at 488 nm using an argon laser line. Fluorescence was measured in channel 1 (FL1-H), which detected Alexa Fluor^®^ 488 (Annexin V) and channel 3 (FL3-H), which detected PI fluorescence. All the measurements were repeated six times. Staining of K562 cells by Annexin V and PI was further confirmed by microscopy, at 200X magnification under a bright-field or fluorescence microscope (Nikon Eclipse Ti-S). Photographs were taken using NIS-Elements BR 3.22.07 software (Nikon Instruments Inc. USA).

### Caspase activity assay

Activation of caspases 3/7, 8, and 9 in the MnSOD-3WT–treated K562 cells (5 × 10^3^ / well) was measured with the appropriate Caspase-Glo assay. Following incubation with MnSOD-3WT (20 µg, 0.56 µM) (24, 48, and 72 h) in 96-well white opaque plates under serum-starved conditions, the cells were allowed to equilibrate for 30 min at room temperature. The respective Caspase-Glo reagent (100 µL) was added to each well and the contents of the wells were mixed for 1 min at 300 rpm. The plates were then incubated at room temperature for 2 h. The luminescence of each sample was measured using a Mithras LB 940 Multimode Microplate Reader at an integration time of 0.5 s per well. Each experiment was performed in triplicate.

### Statistics

For mammalian cell culture experiments, data were expressed as the mean ± SEM. The statistical analysis involved a comparison of the untreated control and the treated cells for each of the individually assayed caspase, hence the Student’s *t* test was applied to each pair of samples assayed. The data collected for the cell viability assays were assessed using the one-way ANOVA and Dunnett’s tests were performed for post hoc multiple comparisons. In all cases, statistical significance was defined as *p* < 0.05 (*), *p* < 0.01 (**), or *p* < 0.001 (***). Statistical analysis was carried out using Graphpad Prism^®^ 6.0 software (USA).

## Results

### The active site of H30N forms a hexa coordinate metal center

**Fig. 1:**
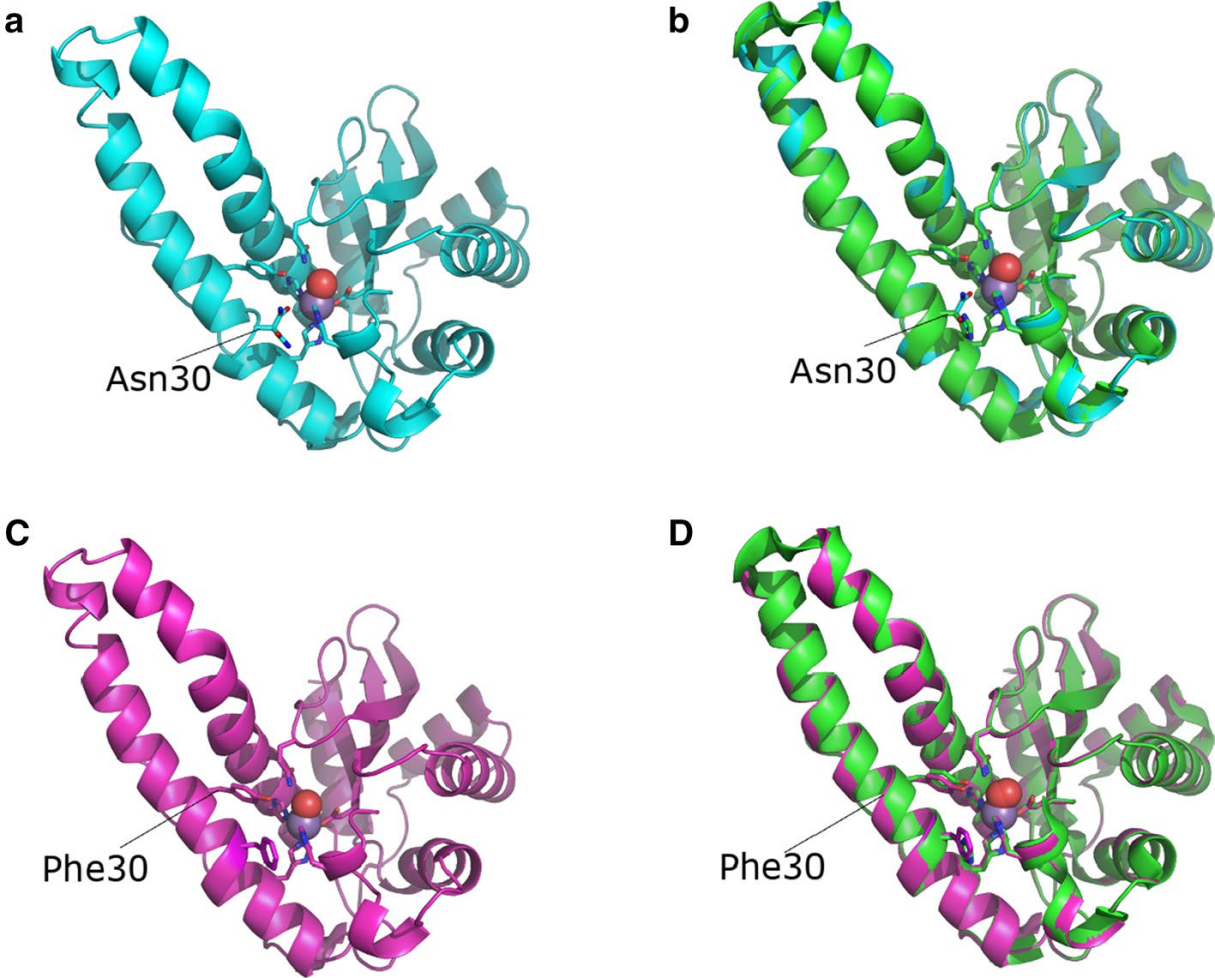
Structures of the manganese superoxide dismutases used in this study. Secondary structure cartoon representations are shown with the manganese ion as a purple sphere and the active-site water, W1, as a red sphere. Ligands to the metal are shown as sticks. **a** The overall fold of the H30N mutant (cyan) with N30 indicated. **b** Superimposition of H30N (cyan) with wild type (green). RMSD 0.204 Å (2684 atoms). **c** H30F (magenta) is depicted as in **a** with F30 indicated. **d** Superimposition of H30F (magenta) with wild type (green). RMSD 0.241 Å (2570 atoms)

Structural and biophysical analyses of the H30N protein support the presence of a hexa coordinate center in the active site of the mutant enzyme. The crystals belong to the space group P4_1_2_1_2, with unit-cell parameters of *a* = *b* = 82.5 Å, *c* = 135.2 Å and structural data were determined at a resolution of 1.52 Å. The asymmetric unit consists of two subunits that are designated A and C. The tetrameric structure, with its dimer of dimers quaternary assembly, is very similar to that of both the native *C. elegans* MnSOD and human MnSOD as are its α-helical hairpin N-domain and α/β C-domain (Trinh et al. 2008) (Fig. 1).

The data collection and refinement statistics for the 100 K H30N and H30F structures are presented in Table 1.

**Table 1:** Crystallographic summary for the structures of H30N (100 K) (pdb: 6S0D) and H30F (100 K) (pdb: 6QZM)

	H30N	H30F
Resolution range (Å) ^a^	58.32–1.52 (1.56–1.52)	40.74–1.60 (1.64–1.60)
Space group	*P*4_1_2_1_2	*P*4_1_2_1_2
Unit-cell parameters (Å)	*a* = *b* = *82.5,* *c* = *135.2*	*a* = *b* = *81.49*, *c* = *138.66*
No. of observed reflections	460,372 (7104)	59,113
No. of unique reflections	66,925 (3872)	59,113
Data redundancy	6.6 (1.8)	4.0
< *I/σ(I)* > ^a^	1.4 (1.5)	4.0 (1.5)
Completeness (%)^a^	96.5 (71.4)	100.0 (100.0)
*R*_merge_ (%)^a,b^	6.9 (58.8)	6.7 (62.8)
*R*_pim_ (%)	3.6 (57.7)	1.9(17.3)
*CC*_*1/2*_	0.99 (0.62)	0.999 (0.934)
Refinement statistics		
Resolution range for refinement (Å)	58.32–1.52	40.74–1.60
*R* factor (%)	17.0	18.8
*R*_free_ (%)^d^	21.9	20.6
No. of protein non-H atoms	3131	3128
No. of water molecules	295	369
No. of manganese ions	2	2
No. of sulfate atoms	7	3
R.m.s.d bond lengths (Å)^ξ^	0.010	0.012
R.m.s.d bond angles (˚)^c^	1.4	1.3
Average overall *B* factor (Å^2^) Ramachandran analysis, the percentage of residues in the regions of plot (%)^e^	34	18
Favored regions^e^ Outliers	97.3 0	97.7 0

^a^Values given in parentheses correspond to those in the outermost shell of the resolution range.

^b^$$R_{{merge}} = {{\sum\nolimits_{{hkl}} {\sum\nolimits_{i} {\left| {I_{i} \left( {hkl} \right) - \left\langle {I\left( {hkl} \right)} \right\rangle } \right|} } } \mathord{\left/ {\vphantom {{\sum\nolimits_{{hkl}} {\sum\nolimits_{i} {\left| {I_{i} \left( {hkl} \right) - \left\langle {I\left( {hkl} \right)} \right\rangle } \right|} } } {\sum\nolimits_{{hkl}} {\sum\nolimits_{{}} {I_{i} \left( {hkl} \right)} } }}} \right. \kern-\nulldelimiterspace} {\sum\nolimits_{{hkl}} {\sum\nolimits_{{}} {I_{i} \left( {hkl} \right)} } }}$$.

^c^$$R_{{pim}} = \sum\nolimits_{{hkl}} {\left\{ {1/[N(hkl) - 1]} \right\}^{{1/2}} {{\sum\nolimits_{i} {\left| {I_{i} \left( {hkl} \right) - \left\langle {I\left( {hkl} \right)} \right\rangle } \right|} } \mathord{\left/ {\vphantom {{\sum\nolimits_{i} {\left| {I_{i} \left( {hkl} \right) - \left\langle {I\left( {hkl} \right)} \right\rangle } \right|} } {\sum\nolimits_{{hkl}} {\sum\nolimits_{i} {I_{i} \left( {hkl} \right)} } }}} \right. \kern-\nulldelimiterspace} {\sum\nolimits_{{hkl}} {\sum\nolimits_{i} {I_{i} \left( {hkl} \right)} } }}}$$.

^d^*R*_free_ was calculated with 5% of the reflections set aside randomly.

^e^Ramachandran analysis using the program MolProbity (Chen et al. 2010)

**Fig. 2:**
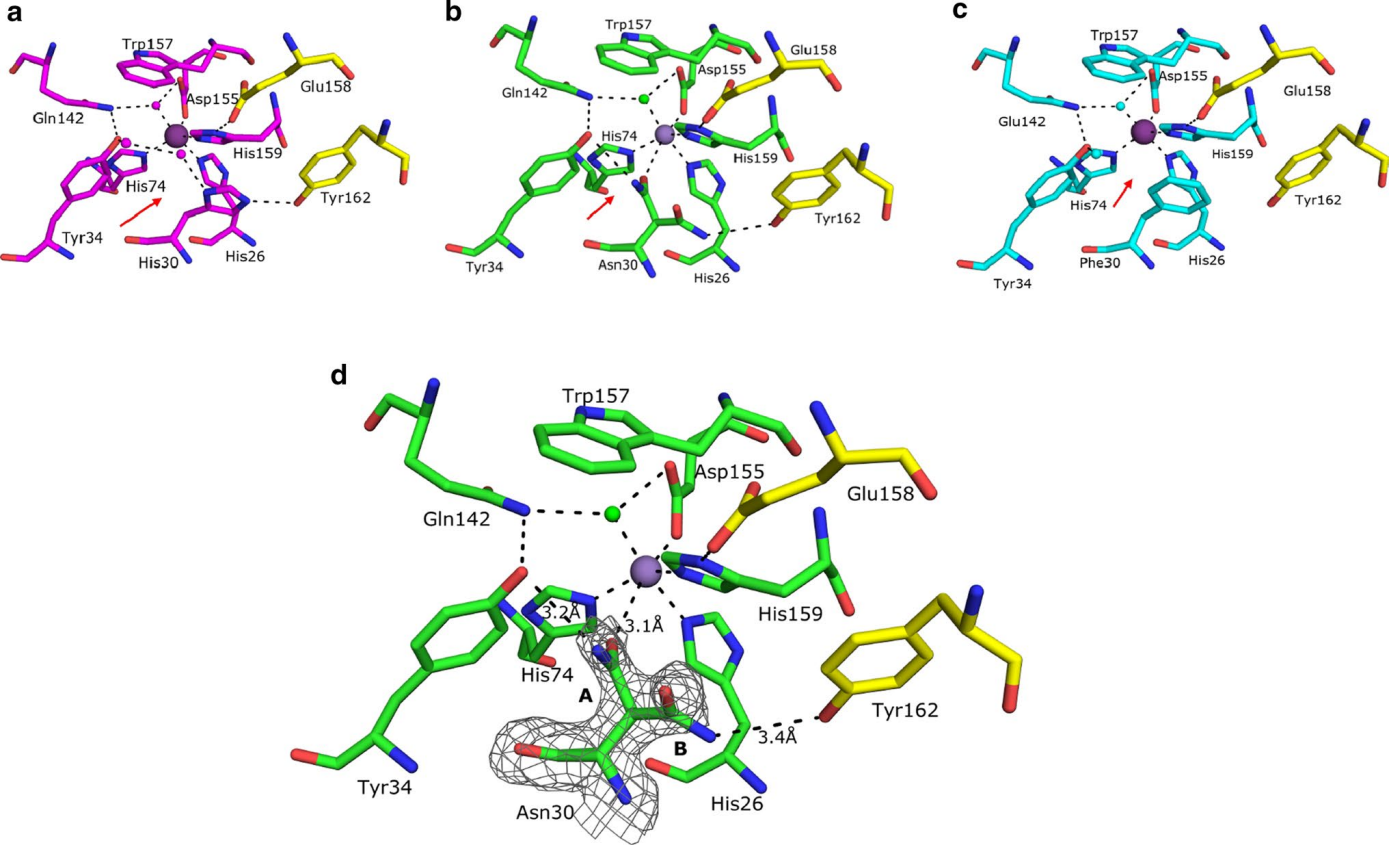
Active sites of **a** MnSOD-3, **b** H30N, and **c** H30F, respectively, with RMSD = 0.25 and bonding according to the H30N active site. Amino acid residues and waters are depicted as magenta, green, and cyan for MnSOD-3, H30N, and H30F, respectively. The yellow residues labeled Glu158 and Tyr162 are from the other subunit of the biological dimer. The red arrows indicate the direction of the solvent in each active site d) Structure of the H30N active site. The hydrogen-bonding network is illustrated as viewed from the approximate direction of substrate access. Both rotamers A and B of Asn30 are depicted, labeled, and surrounded by electron density (σ level = 1). The manganese ion and the solvent molecules are shown as purple and green spheres, respectively. Bonding is illustrated by black dashes (pdb code: 6S0D)

In the wild-type protein, the active site adopts the five coordinate trigonal bipyramidal configuration, common to other MnSODs (Trinh et al. 2008), and the ligands being His26, His74, Asp155, His159, and a water/hydroxyl molecule (W1). His30 forms part of the hydrogen-bonded network by interacting with Tyr34 via two water molecules (Tyr34OH-W2-W3-His30NE2; 2.66 Å, 2.88 Å, and 2.79 Å, respectively) and Tyr162 (2.7 Å) from the other subunit of the biological dimer (Hunter et al. 2015) (Fig. 2a).

Analysis of the H30N active site shows no significant perturbation of the metal ligand interactions, specifically the interactions of His26, His74, His159, and Asp155 with the metal (Fig. 2b). Interestingly, the Asn30 residue can adopt two alternative rotameric positions (Fig. 2d). This was observed in the electron density of each of the active sites making up the asymmetric unit and in other lower resolution H30N crystal structures that we resolved. The designated Asn30 rotamer A is in a proximal conformation relative to the manganese center while rotamer B is distal to the manganese (Fig. 2d). When in the proximal conformation, Asn30OD1 is 3.1 Å away from the manganese center and is capable of acting as a sixth metal ligand. It is worth noting, however, that this distance between OD1 and Mn is a somewhat greater than the typical distance between the other metal ligand interactions taking place in the active site, which range between 2.0 and 2.2 Å. This may suggest a weaker interaction and would explain the presence of two alternate conformations for the Asn30 side chain. Otherwise, electron density would be expected for only the proximal conformation. In this position Asn30ND2 can also hydrogen bond directly with Tyr34OH (3.3 Å), with no intermediate water molecules. The narrow gap between these two residues at the mouth of the solvent funnel will undoubtedly hinder the flow of water and superoxide molecules to the active site, and may retard the release of the hydrogen peroxide product. The interaction between Asn30 and Tyr162 (from the neighboring subunit) is no longer feasible with rotamer A, at a distance of 6.4 Å (Y162OH to N30ND2).

ND2 of the distal rotamer B is rotated 128.5° away from rotamer A ND2, a movement that places it 5.7 Å away from the manganese center and results in a five coordinate trigonal bipyramidal configuration of the active site as seen in wild-type enzymes. In position B, it is now capable of weakly hydrogen bonding with Tyr162 (3.4 Å) from the neighboring subunit. The distance between Asn30ND1 and Tyr34OH is similar to that between His 30ND1 and Tyr34OH in the native protein (5.7 Å and 5.5 Å, respectively). Surprisingly, an extensive, uninterrupted hydrogen-bonding network is observed between the distal Asn30 and the Tyr34, which would support the proton relay to the metal center. As opposed to what is observed in MnSOD-3WT, where two waters mediate bonding between Tyr34 and His30, no waters are observed between Tyr34 and Asn30 (Fig. 2a, b). It may be envisaged that the side chain of Asn30 is highly mobile due to the presence of two occupancies in the electron density, and that this may be caused by the attraction of Asn30 to the metal center. A decrease in enzyme activity, therefore, would result when the proximal rotamer obscures the active site and blocks substrate access to the metal. Replacement of the histidine by phenylalanine in H30F interrupts the hydrogen bond interaction that the His30 normally makes with both Tyr162 and the solvent molecule network which bridges to Tyr34 (Fig. 2c). The Tyr34OH is displaced by 0.49 Å in the direction of Phe30 when compared to Tyr34OH in MnSOD-3WT and the gamma carbon (CG) of Phe30 is 1.39 Å closer to Tyr34OH than the CG side chain of His30 in MnSOD-3WT. This reduces the entrance to the solvent-filled funnel, which is located between residues Tyr34 and Phe30, by 1.5 Å in H30F. Limited access to the superoxide substrate due to the steric effect of phenylalanine may explain the negligible activity exhibited by this particular protein.

**Fig. 3.**
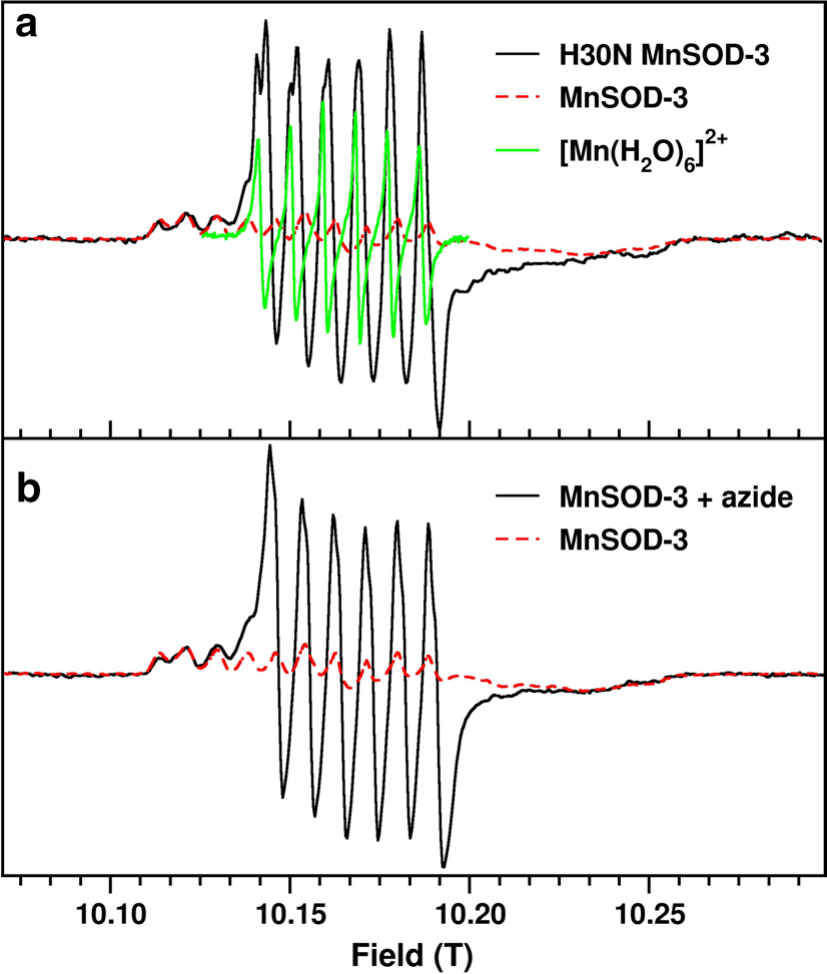
285 GHz HFEPR spectra **a** 285 GHz HFEPR spectra of MnSOD-3WT and H30N. The HFEPR spectrum of H30N (black) is superimposed on that of MnSOD-3WT (dashed red). The H30N spectrum is composed of three components, one similar to the MnSOD-3WT spectrum that corresponds to penta-coordinate Mn(II) centers and two six-lines component arising form hexa-coordinated centers. One of these has the spectrum as that of [Mn(H2O)6]2+. **b** The HFEPR spectra of MnSOD-3WT in the presence and absence of 50 mM azide. The HFEPR spectrum of MnSOD-3WT after azide removal is depicted in red dotted lines

**Fig. 4:**
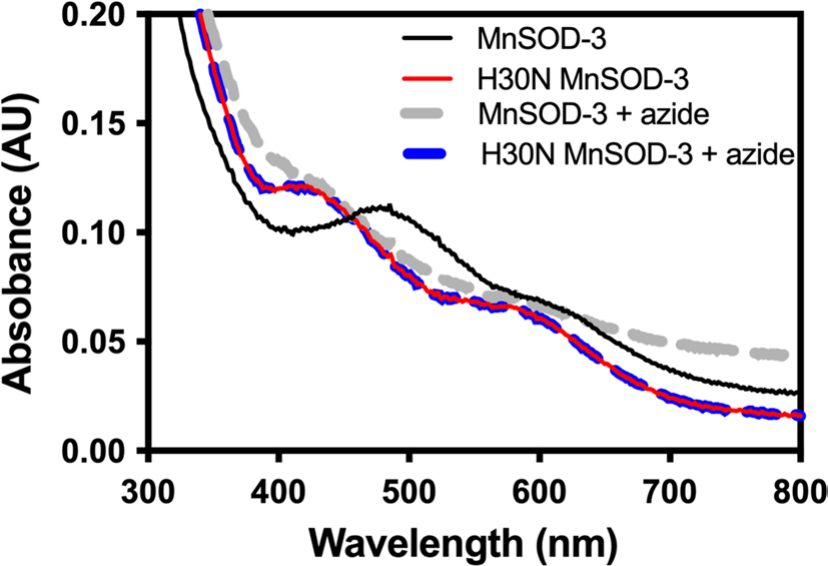
Absorption spectra of oxidized MnSOD proteins in the absence and presence of 100 mM azide. The characteristic Mn(III) peak at 478 nm is present in the visible absorption spectrum of MnSOD-3, while a maximum at 420 nm is observed for the H30N mutant

Further characterization of H30N by 285 GHz high-field electron paramagnetic resonance (HFEPR) confirmed the occurrence of a hexa coordinate center (Figs.3a and b). The HFEPR spectra of MnSOD-3WT resemble that of other MnSODs (Fig. 3a) (Un et al., 2004; Tabares et al. 2010)). The broad spectrum between 10.11 and 10.26 T arises from *m*_*s*_ = -1/2 1/2 spin transition of the Mn(II) center (*S* = 5/2). The fine structures arise from the hyperfine interaction of the ^55^Mn (*I* = 5/2) nucleus and six unpaired electrons. The shape and width are the consequence of the large GHz Mn(II) zero-field interaction of the penta-coordinate Mn(II) center in SODs. This larger zero-field interaction is a characteristic of Mn(II)SODs and its specific magnitude highly correlates with the Mn or Fe metal specificity (Barwinska-Sendra et al. 2020). Upon addition of the azide, a narrower six-line component (from 10.13 to 10.20) was observed. As we have seen, (Tabares et al. 2006), this change corresponds to a large reduction of the zero-field interaction to about 1.4 GHz as a consequence to the azide binding and is indicative of the formation of the hexa-coordinated center (Fig. 3b). As with other SODs, azide binding to MnSOD was only partial resulting in a mixture of azide-free penta-coordinated centers and azide-bound hexa-coordinated centers (Tabares et al. 2006). The HFEPR spectrum of H30N was similar to that of MnSOD-3WT with azide (Fig. 3a and b). This indicates the presence of penta- and hexa-coordinate centers in the resting state of the protein. We have shown that these hexa-coordinated centers can be obtained by water coordination to the Mn(II) center and observed an increase in water occupancy when in the Y34F mutant of *E. coli* MnSOD (Tabares et al. 2007). Therefore, the HFEPR spectra of H30N show that in the resting state the protein is in a composition of an approximately 1:1 mixture of normal penta-coordinate centers and water-bound hexa-coordinated centers. A much smaller six-line Mn(II) spectrum similar to that of non-specific [Mn(H_2_O)_6_]^2+^ was also detected. The concentration of species giving rise to the signal was estimated to be < 5% of the total Mn(II) concentration based on double integration analysis as we have shown previously) concentration based on double integration analysis as we have shown previously. Addition of azide showed negligible effect on the H30N spectrum (data not shown). The predominant oxidation state in MnSOD-3WT is Mn(III), which gives the characteristic peak at 480 nm in the visible absorbance spectrum (Fig. 4). The addition of 100 mM azide to the protein caused a blue shift to 420 nm (Fig. 4). Azide is a substrate analog that binds to the manganese cofactor forming a hexa coordinate center (Hunter et al. 2015). The visible spectrum of H30N lacks the 480 nm peak, and yet it has a peak at 420 nm (Fig. 4).

Many of our protein purifications contained both iron and manganese in varying ratios and we never managed to isolate one over the other, even in minimal media. It, however, appears that the H30N mutant has a higher selectivity for iron than the wild type when prepared in minimal media. When prepared in 2TY media, the maximum manganese occupancy observed was 0.62. When prepared in minimal media, H30N had an iron occupancy of 0.75. In addition, the samples tend to be yellow in color and their visible spectrum is suggestive of iron. In contrast, the uptake of manganese by the MnSOD-3WT protein was 100%. The protein used in the H30N crystal structures contained 0.7% Fe and 0.3%Mn when later analyzed. The metal of course cannot be determined from the X-ray structure which most likely is a hybrid in favor of iron.

### Mutation of histidine 30 affects enzyme activity and stability

**Fig. 5:**
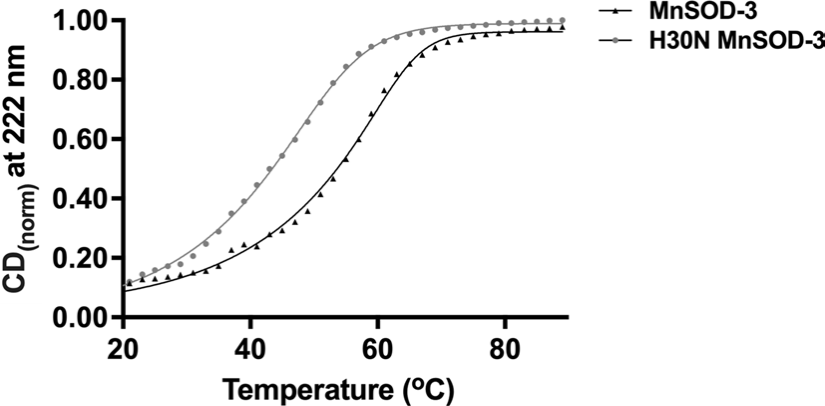
Temperature stability normalized CD curves for MnSOD-3WT and H30N protein**.** The complete temperature unfolding profiles at 222 nm for MnSOD-3WT and H30N are depicted by an asymmetric (five parameter) least squares fit to calculate the melting temperature. The *T*_m_ of MnSOD and H30N were calculated to be 52 and 42 °C, respectively

As part of our study of SOD catalysis, we replaced the histidine with asparagine (Fig. 2). Analysis of H30N shows that at 25 °C the protein retains 22% the activity of the wild type (550 U mg^−1^ vs 2500 U mg^−1^). The mutation also affects the conformational stability of the protein as the plot of the change in CD intensity at 222 nm with temperature (Fig. 5) shows a reduction of 10 °C in the midpoint of the unfolding transition or T_m_ for H30N (52 °C MnSOD-3WT, 42 °C H30N). This instability is also reflected in a more pronounced loss of SOD activity both over time and with an increase in incubation temperature. Thermal inactivation assays whereby protein samples were incubated for 10 min at temperatures ranging from 25 to 85 °C gave a T_50_ of 59 °C for MnSOD-3WT and 48 °C for H30N. The reduced enzymatic activity of the H30N protein at room temperature may also be due to the movement of the Asn30 rotamer to a position where it blocks access of the superoxide substrate to the active site as observed in the resolved structure.

### Molecular dynamic simulations

Molecular dynamics (MD) simulations of the H30N mutant protein were performed to obtain structural information where experimental techniques such as X-ray crystallography, are inapplicable. MD was conducted at 300 K as lower temperatures would artificially slow down the movement of the molecule. By performing MD simulations, we can get an indication of movement and subsequent interactions between the Asn30, other neighboring amino acids such as Tyr34 and inner-sphere waters and the metal cofactor which may not be visible in the X-ray structure of the molecule. The MD simulations that were carried out allowed the motion of the H30N protein to be simulated in defined conditions on the basis of classical molecular dynamics, using the structure including rotamer A of Asn30 as a starting point. As both manganese and iron ions were detected in the H30N protein by GF-AAS and ICP-MS, analysis, molecular dynamic simulations of the H30N mutant were performed using Mn and Fe parameters. Two simulations of 80 ns were performed to investigate the effect of the mutation and any changes that may be due to the manganese or iron metal cofactor.

**Fig. 6:**
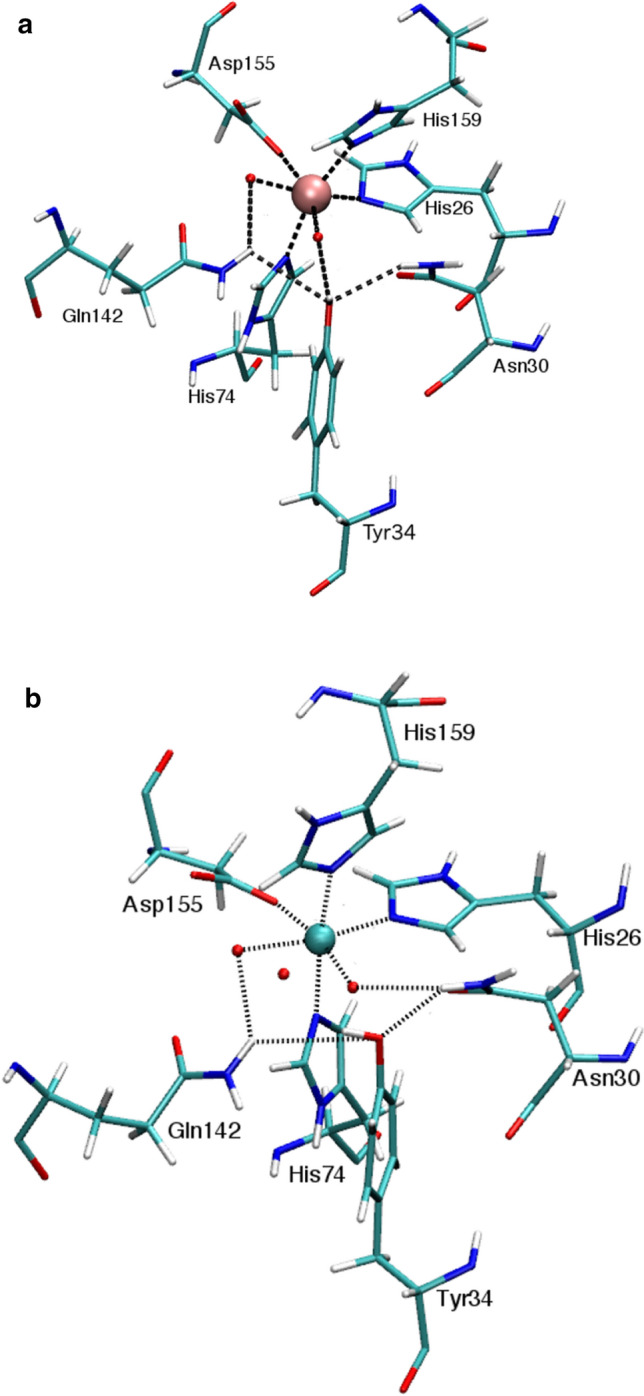
H30N active sites with **a** manganese and **b** iron cofactors observed from molecular dynamic simulations. These snapshots of the protein trajectory reveal the way in which the hydrogen-bonding network is maintained in the active sites containing either Mn or Fe. In **a**, the hydrogen-bonding network was maintained for 18% of the simulation. In **b**, the hydrogen-bonding network was maintained for 50% of the simulation. Red spheres indicate waters. Pink and cyan spheres indicate manganese and iron, respectively. Hydrogen bonding is illustrated as black dashes

The presence of a well-connected hydrogen-bonding network that spans from the manganese, water, Gln142, Tyr34, and Asn30, was observed in only 18% of the frames in the simulation trajectory when H30N had manganese as its cofactor (Fig. 6a). On the other hand, the network of hydrogen bonding was observed in 50% of the frames constituting the simulation trajectory of the H30N, which contained iron as its cofactor (Fig. 6b). In this case the network extended from the iron cofactor, moving to water, Gln142, Tyr34, Asn30, water, and ending back with iron. In contrast, no mediating waters were observed in the crystal structure determined for H30N, apart from the water molecule coordinated to the metal cofactor. MD indicates that a change in the metal cofactor of the protein could actually alter the motions of the active-site residues and water molecules such that the hydrogen-bonding network could be maintained more regularly in the H30N simulation containing iron.

### Exogenous MnSOD and H30N have opposing effects on the cell viability of K562 cells

**Fig. 7:**
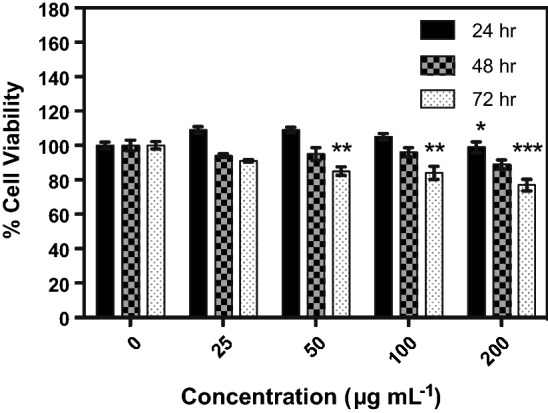
Exogenous MnSOD-3WT treatment of K562 cells. Percentage cell viability of K562 cells as estimated by CellTiter Glo proliferation assay in 96-well plates with serum-free media, following 24, 48, and 72 h exposure to varying amounts of MnSOD-3WT. Results represent mean ± SEM, *n* = 3. Statistical significance was defined as *p* < 0.05 (*) or *p* < 0.01 (**)

The K562 cells were seeded at 5 × 10^3^/well in serum-free RPMI (100 µL) with increasing amounts of MnSOD-3WT and H30N to investigate the effect these SODs may have on the cell viability. Incubation of the cells with varying amounts of MnSOD-3WT for 24, 48 and 72 h showed a statistically significant decrease in viability in both a time-dependent and a dose-dependent manner when compared to the untreated cells (Fig. 7). Incubation for 72 h in the presence of 50 µg ml^−1^ of MnSOD caused a 15% loss in viability. This effect was dose-dependent and was more pronounced at higher protein concentrations, exhibiting a 33% decrease with 200 µg ml^−1^ of the protein. The latter parallels the loss in viability caused by incubation with 10 µM H_2_O_2_. To ensure that the observed effect was not just a consequence of osmotic stress due to the supplemented protein, a parallel experiment was performed with the relatively inactive H30F mutant. At the given concentrations, the H30F protein had no effect on the cell viability thereby supporting that the loss of viability observed for MnSOD-3WT was related to its superoxide dismutase activity (S1). The addition of only Tris.Cl buffer also had no effect on cell growth (S2). The effect of catalase on the cell viability was also examined. Treatment of K562 cells with 30 U catalase improved cellular viability by 65% after 48 h of incubation. This indicates that hydrogen peroxide is present in serum-free media, in amounts that curtail proliferation, demonstrating that the removal of hydrogen peroxide by catalase does suppress apoptosis in the MnSOD-treated cells (Halliwell et al. 2000).

**Fig. 8:**
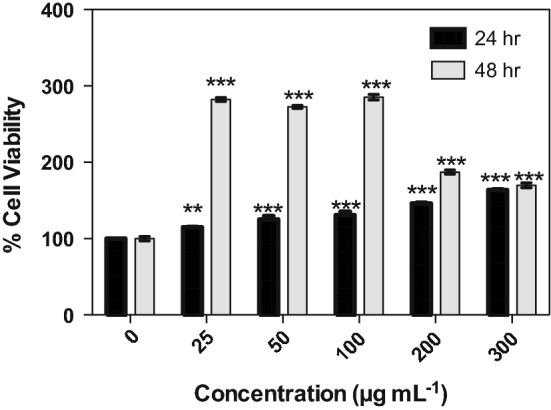
Effects of the H30N on K562 cell proliferation by CellTiter-Glo^®^ test. Various concentrations of H30N were added to the medium and incubated for 24 and 48 h, respectively. Any measurements taken after 72 h incubation resulted in substantial cell death due to previous cell overgrowth. Data were presented as means ± SEM, *n* = 3. Statistical significance was defined as *p* < 0.01 (**) or *p* < 0.001 (***)

When we treated the K562 cells with H30N we were surprised to observe an increase in cell viability. The greatest increase of 282% ($\pm 2.61)$, 272% ($\pm 1.96)$, and 285% ($\pm 3.75)$ occurred after 48 h when cells were supplemented with 25 µg ml^−1^, 50 µg ml^−1^, and 100 µg ml^−1^ of the protein, respectively (Fig. 8). Untreated cells or cells treated exogenously with H30F or Tris.Cl exhibited no significant changes. After 72 h, cells showed a decrease in viability that may be attributed to a depletion of nutrients and optimal growth conditions (results not shown).

### Exogenous MnSOD triggers apoptosis in K562 cells

**Fig. 9:**
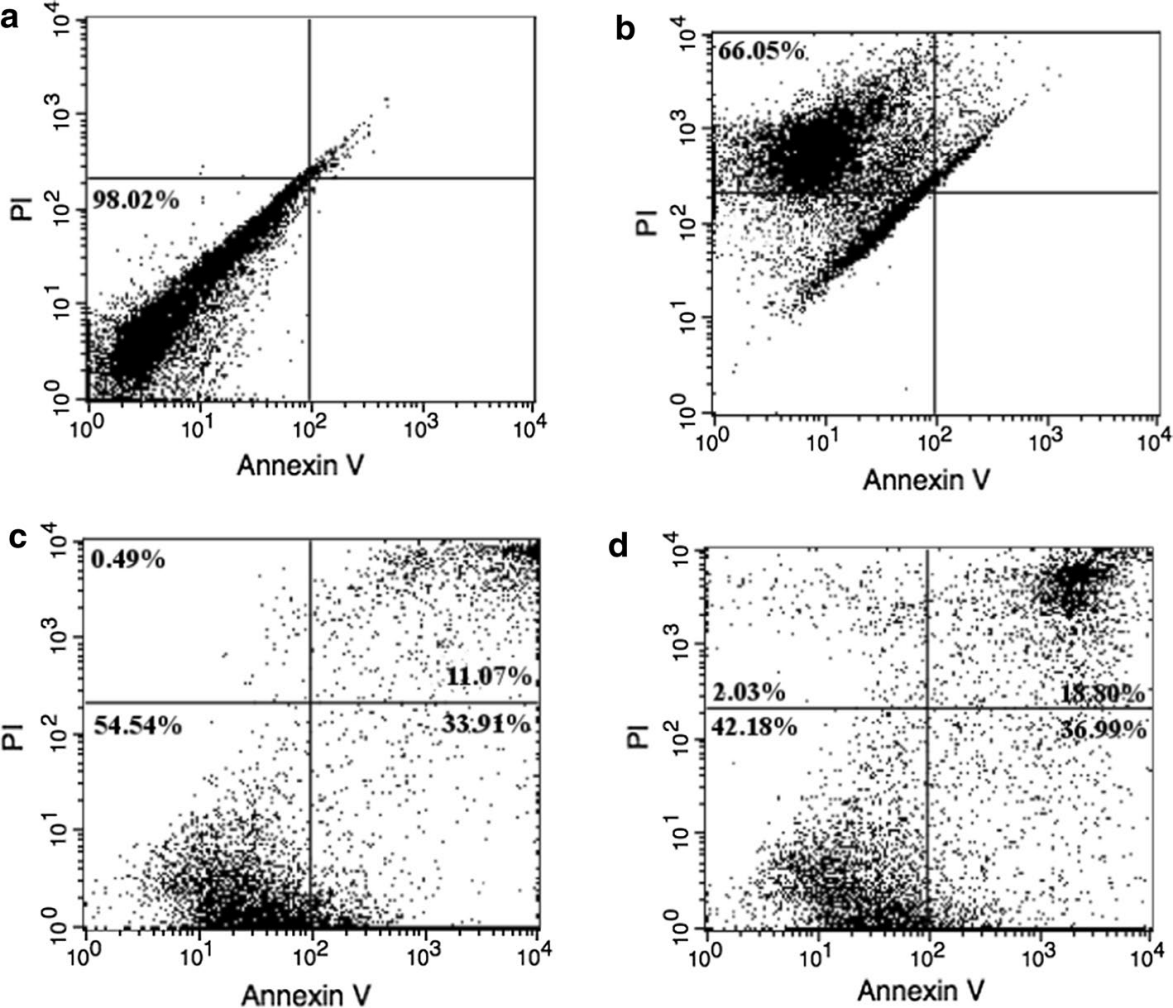
Analysis of apoptosis and necrosis in MnSOD-3WT-treated K562 cells using flow cytometry. **a** Unstained viable K562 cells, acting as a negative control. **b** PI stained necrotic K562 cells, acting as a positive control. **c** Untreated K562 cells double stained with Annexin V and PI (*n* = *6).* **d** MnSOD-3WT treated (200 μg mL^−1^) K562 cells double stained with Annexin V and PI (*n* = *4).*

**Fig. 10:**
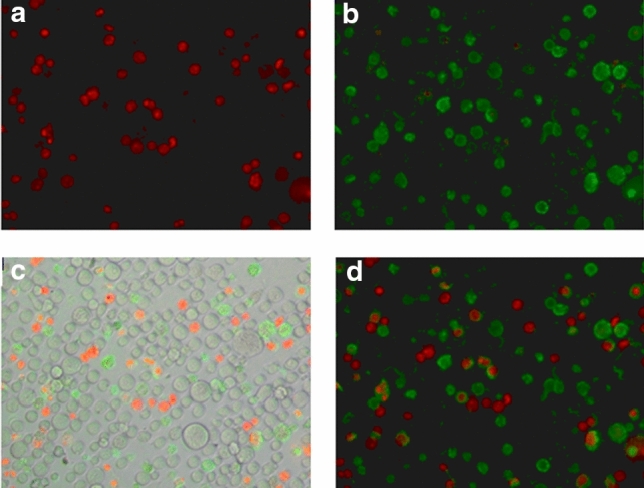
Apoptosis assessment of MnSOD-3WT-treated K562 cells by representative bright-field and fluorescence microscopy images (× 200)**.** **a** Necrotic cells stained by PI only (red). **b** Apoptotic cells stained by Annexin V only (green). **c** Bright-field image illustrating Annexin V/PI stained cells. **d** Merged fluorescence image illustrating Annexin V/PI stained cells

Using the 48-h sample of MnSOD-3WT-treated cells, we tested whether the observed loss of viability was related to apoptosis or necrosis. The fluorophore-conjugated Annexin V identifies apoptotic cells by binding to phosphatidylserine that moves from the inner to the outer leaflet of the plasma membrane in apoptotic cells. Necrotic cells, on the other hand, are permeable to propidium iodide (PI) that binds to nuclear material and stains the cells red thereby distinguishing necrotic cells from both viable and apoptotic cells. Unstained viable K562 cells were used as a negative control (Fig. 9a). PI stained K562 cells were used as a positive control for necrosis (Fig. 9b). Benzo(a)pyrene-induced apoptotic cells served as the positive control to define gating and quadrant parameters for flow cytometry. The populations of cells that were observed following MnSOD-3WT treatment included cells that were viable and not undergoing apoptosis in the lower left of quadrant (Annexin V and PI negative, Fig. 9c); cells undergoing early apoptosis, observed in the lower right of the quadrant (Annexin V positive and PI negative, Fig. 9d); cells in a late stage of apoptosis observed in the upper right quadrant (Annexin V positive and PI positive) and dead cells observed in the upper left corner (Annexin negative and PI positive). The percentage of cells in the late apoptotic and necrotic stage was 6% higher in the treated K562 cells compared to the untreated cells while the viability of the treated cells was decreased by 12%, with respect to the untreated control. These results were statistically significant and compared well with the loss in cell viability measured by the CellTiter-Glo^®^ assay (Fig. 7). Progression of apoptosis was also confirmed by bright field and fluorescence microscopy (Fig. 10), following Annexin V/PI double staining of the treated cells. The bright-field images show the healthy Annexin V and PI negative cells. The cells in early apoptotic stages stained only green due to Annexin V, while the necrotic cells were stained only red by PI. The late apoptotic cells appear stained by both Annexin V and PI (green and red). The larger number of cells in a late apoptotic stage in treated cells suggests a greater induction of apoptosis due to MnSOD-3WT treatment.

### MnSOD-3WT treatment causes differential caspase activation

**Fig. 11:**
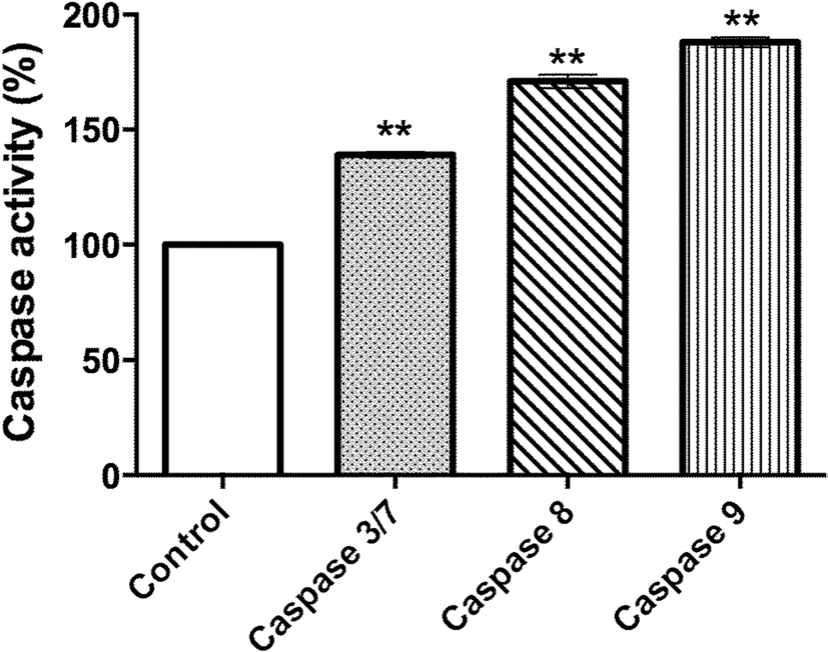
Cellular caspase 3/7, 8 and 9 activity over 72 h in MnSOD-3WT treated cells. Caspase activity in MnSOD-3WT treated cells was expressed as a percentage of the caspase activity measured in the untreated control cells. Results shown represent mean ± SEM, *n* = 3. Statistical significance was defined as *p* < 0.05 (*) or *p* < 0.01 (**)

We investigated the activation of caspases 3/7, 8, and 9 to further confirm that the observed loss in cell viability was in fact due to the onset of apoptosis. Significant increases in caspase 3/7, 8, and 9 activities were recorded after 72 h of MnSOD-3WT treatment (Fig. 11). Relative to the untreated samples, the greatest increase was recorded for the initiators of apoptosis, caspase-9 (88%) and caspase-8 (71%) and to a lesser extent, the executors of apoptosis caspase 3 and 7 (31%). Our results, therefore, suggest that the hydrogen peroxide product of the exogenous SOD reaction stimulates both the extrinsic (via surface death receptor) and the intrinsic (via mitochondria) apoptotic pathways (Siegel et al. 2003). Both extrinsic and intrinsic pathways converge to activate caspases 3 and 7 that in turn, transmit downstream signals for cell-wide disassembly.

## Discussion

Results indicate that altering the His30 gateway residue to Asn30 has changed the physicochemical properties, the active site structural coordination and the effect that MnSOD-3WT can exert on the proliferation of K562 cells when added exogenously.

One of the main changes induced by the single-amino acid substitution of histidine by asparagine was a reduction in the incorporation of manganese in the active site and an increase in that of iron. Such an occurrence was also observed in the Q142H mutant of *C. elegans* MnSOD, whereby substitution of the active-site residue at position 142 from glutamine to histidine also changed the metal selectivity from manganese to iron (Hunter et al. 2018). Preliminary results also revealed that metal selectivity is altered in the H30N mutant of human MnSOD (unpublished). However, it was observed that when histidine replaced glutamine 142 in the active site, the enzyme retained 50% of its activity and became cambialistic for its metal cofactor exhibiting very similar specific activity with either manganese or iron. Although the H30N mutant protein also revealed the spontaneous uptake of Fe in addition to Mn, enzyme activity was drastically reduced to 22% in this case. The decrease in enzyme activity of H30N was also accompanied by a decrease in the thermal melting temperature (42˚C) when compared to MnSOD-3WT (52˚C). The loss of the interaction between the Asn30 and Tyr162 from the neighboring subunit) may be a contributing factor to the loss of conformational stability observed by CD and thermal inactivation of enzyme function.

Spectrophotometric, HFEPR, and X-ray crystallography experiments together with MD simulations of H30N with an iron cofactor all consolidate the fact that MnSOD-3WT undergoes a change in its active-site geometry upon the single mutation from histidine to asparagine at position 30. HFEPR experiments especially highlight the presence of mixed geometry active sites, as the spectrum for the H30N mutant shows the fingerprint feature of typical penta-coordinate manganese center, as well as the six-line spectral feature, which represents octahedral geometries. The absence of a 480 nm peak in the absorption spectrum of H30N also indicates the minimal presence of manganese in the protein, when compared to its wild-type counter-part. The solved X-ray structure and the MD simulation demonstrate the increased mobility that asparagine replacement has brought about in the second sphere of the H30N protein, in order for the protein to maintain a level of functional catalysis. The X-ray structure demonstrates the adoption of two rotamers by Asn30, one of which can directly coordinate with the cofactor to form octahedral active-site geometry. In the X-ray structure, Asn30OD1 proximal position forms the sixth coordination with the manganese. However, in the MD simulation in Fig. 6a the Asn30OD1 is facilitating the sixth coordination via a water molecule to the manganese cofactor. The MD simulation of H30N containing iron as a cofactor also illustrates how Asn30 facilitates the formation of octahedral centers by coordinating with the metal cofactor via an intermediary water molecule.

Although MnSOD protein cannot enter the cell, the hydrogen peroxide product of its reaction is capable of crossing the plasma membrane by diffusion and via aquaporins (Vieceli Dalla Sega et al. 2014). The K562 cells were selected for this study because they are non-adherent cells that produce high levels of superoxide radicals extracellularly, via the activity of their over-expressed membrane-bound NADPH oxidase (de Mendez and Leto 1995). This superoxide may, in turn, serve as substrate for the exogenously supplemented superoxide dismutase.

The drastic changes in active-site geometry in the H30N mutant have also resounded in the observed effect on K562 cells, upon exogenous addition of this protein. One inference that may be derived from the modified active-site structure and activity of H30N together with the signaling properties of hydrogen peroxide would be that H30N has a catalytic mechanism, which generates hydrogen peroxide product at a level, which stimulates cell growth. Davis et al., in fact state that hydrogen peroxide can act as a secondary messenger with the unique effects of stimulating cell proliferation at concentrations of 100 nM to 1 μM (Davis et al. 2004). On the other hand, *C. elegans* MnSOD, is catalytically similar to human H30N (Hunter et al. 2015), as both exhibit a low level of product inhibition. Therefore, both of these enzymes may produce larger amounts of hydrogen peroxide, which result in the anti-proliferative effects observed on K562 cells (Davis et al. 2004). While the presence of MnSOD-3WT caused a dose-dependent decrease in proliferation, interestingly, the presence of both MnSOD-3WT and catalase resulted in a 17% increase in cell proliferation. This highlights the fine balance between hydrogen peroxide-induced proliferation and apoptosis in cellular systems. This was further substantiated by the comparison of the cellular viability in response to SODs with varying activities. This ties in with the apoptosis-inducing character of hydrogen peroxide that is the product of the SOD reaction. The highly active MnSOD-3WT caused a decrease in viability by triggering apoptosis. The H30N protein that has a fifth of the activity of the wild type stimulated cellular proliferation, to an extent similar to that exhibited by the combination of MnSOD-3WT and catalase. The fact that no change in viability resulted from treatment with the inactive H30F protein suggests that the level of hydrogen peroxide produced by MnSOD actually influences the cellular switch between proliferation and apoptosis.

## Supplementary Information

Below is the link to the electronic supplementary material.

Supplementary file 1 (DOCX 248 KB)

## Abbreviations

IPTG: Isopropyl β-d-1-thiogalactopyranoside
H30N: MnSOD-3[H30N]
H30F: MnSOD-3[H30F]
CD: Circular dichroism
MD: Molecular dynamics
HFEPR: High-field electron paramagnetic resonance
SEM: Standard error of the mean
