# Supplementary material for: Substitution of histidine 30 by asparagine in manganese superoxide dismutase alters biophysical properties and supports proliferation in a K562 leukemia cell line
Source: Eur Biophys J. 2021 May 21;50(3-4):571–85. doi: 10.1007/s00249-021-01544-2 (PMC8190026; doi:10.1007/s00249-021-01544-2)
Supplement: Supplementary file 1 — (DOCX 248 KB) [file 249_2021_1544_MOESM1_ESM.docx]

**Supplementary material**

**Substitution of histidine 30 by asparagine in manganese superoxide dismutase alters biophysical properties and supports proliferation in a K562 leukemia cell line**

*Rosalin Bonetta^1,8^, Gary J. Hunter^2^, Chi H. Trinh^3^, Tomasz Borowski^4^, Anthony G. Fenech^5^, Maria Kulp^6^, Leandro C. Tabares^7^, Sun Un^7^, Thérèse Hunter^2^

1 Centre of Molecular Medicine & Biobanking, University of Malta, Malta

2 Department of Physiology and Biochemistry, Faculty of Medicine and Surgery, University of Malta, Malta

3 Astbury Centre for Structural Molecular Biology, Institute of Molecular and Cellular Biology, University of Leeds, Leeds, UK

4 Jerzy Haber Institute of Catalysis and Surface Chemistry, Polish Academy of Sciences, Krakow, Poland

5 Department of Clinical Pharmacology and Therapeutics, Faculty of Medicine and Surgery, University of Malta, Malta

6 Department of Chemistry, Tallinn University of Technology, Tallinn, Estonia

7 Université Paris-Saclay, CEA, CNRS, Institute for Integrative Biology of the Cell (I2BC), 91198, Gif-sur-Yvette, France.

8 Current address: Barts and the London School of Medicine and Dentistry, QMUL, Malta

*Corresponding author. E-mail address: [r.bonetta@qmul.ac.uk](mailto:r.bonetta@qmul.ac.uk)


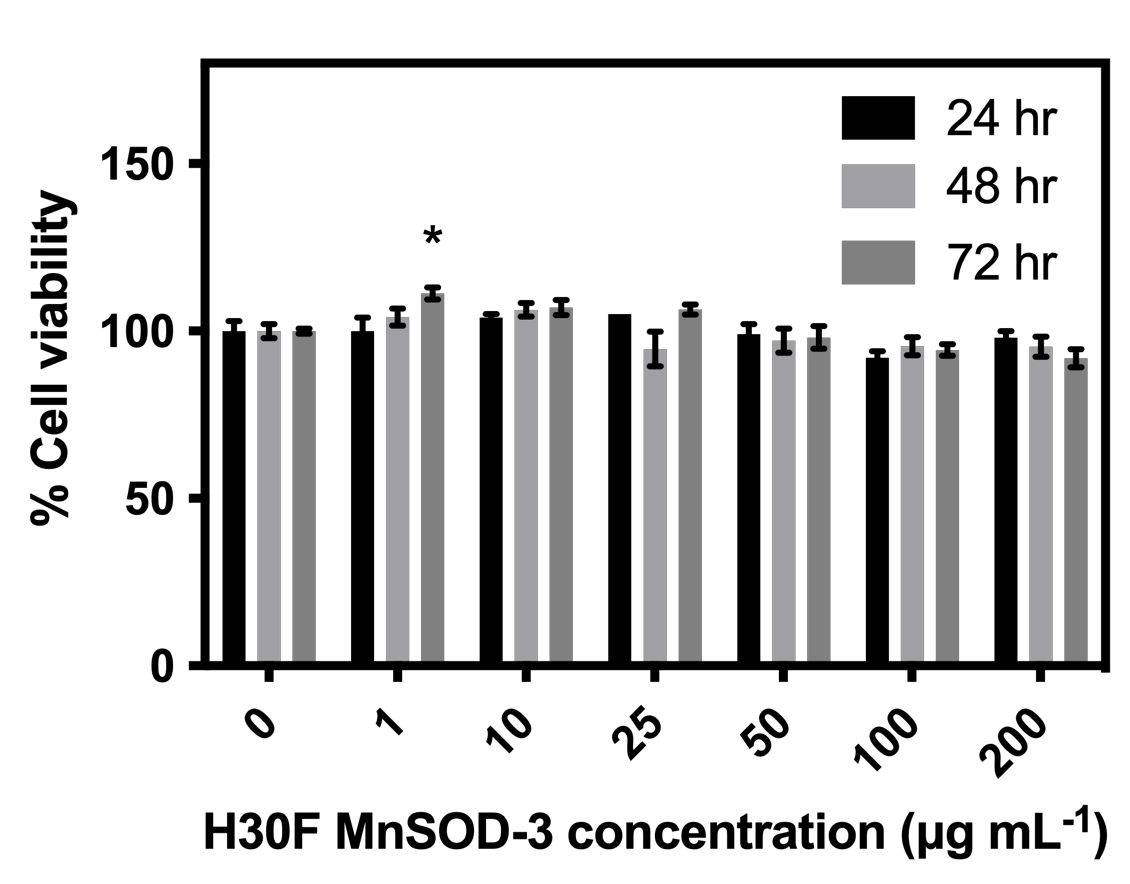


**Figure S1** Exogenous H30F treatment of K562 cells. Percentage cell viability of K562 cells as estimated by CellTiter Glo proliferation assay in 96-well plates with serum-free media, following 24, 48 and 72 hours exposure to varying amounts of MnSOD-3. Results represent mean ± SEM, *n*=3. Statistical significance was defined as *p* < 0.05 (*)


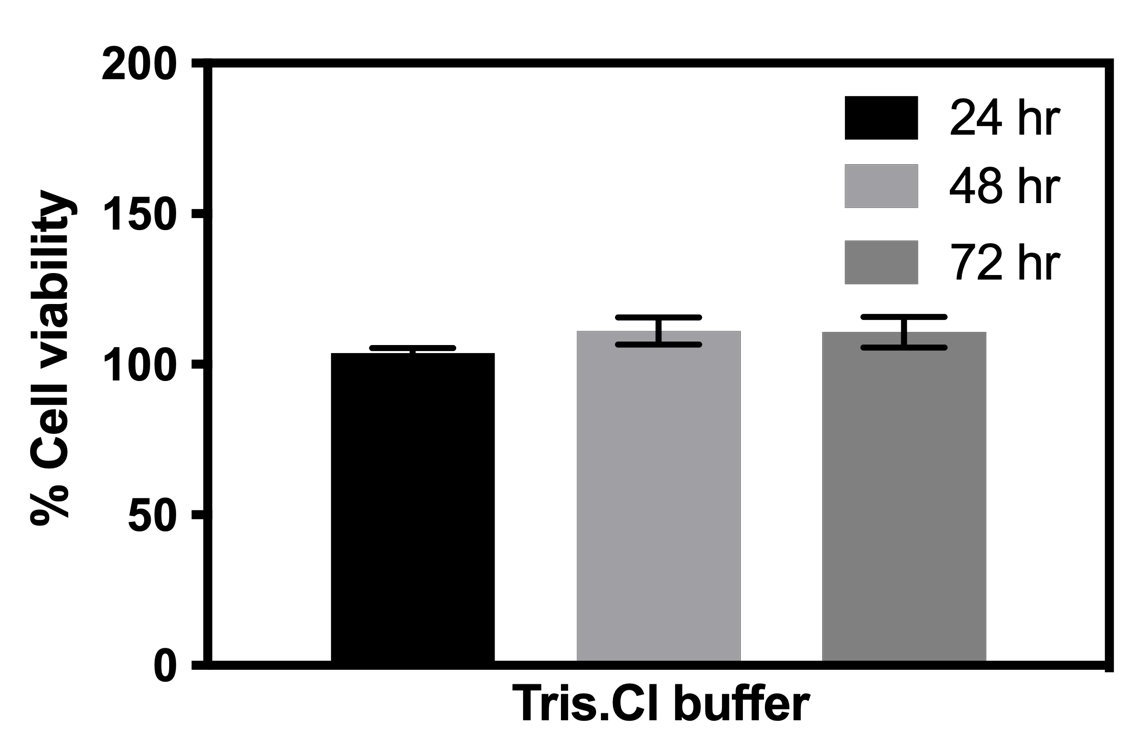


**Figure S2** Exogenous Tris.Cl buffer pH8 treatment of K562 cells. Percentage cell viability of K562 cells as estimated by CellTiter Glo proliferation assay in 96-well plates with serum-free media, following 24, 48 and 72 hours exposure to Tris.Cl buffer in comparison to untreated cells. Results represent mean ± SEM, *n*=6. No statistical significance was observed when compared to the untreated control K562 cells
